# Iron oxide coated hollow poly(methylmethacrylate) as an efficient adsorption media for removal of arsenic from water

**DOI:** 10.1039/d0ra10801d

**Published:** 2021-04-12

**Authors:** Dhiraj Dutta, J. P. Borah, Amrit Puzari

**Affiliations:** National Institute of Technology Nagaland Chumukedima Dimapur 797 103 Nagaland India amrit09us@yahoo.com

## Abstract

Adsorption of arsenic onto iron-based adsorption media has been established as a convenient method for the removal of arsenic from contaminated water. The study describes the efficiency of iron oxide coated hollow poly(methyl methacrylate) microspheres (FHM) as an adsorptive media for the removal of arsenic from water. Hollow poly(methyl methacrylate) microspheres (HPMM) were synthesized by solvent evaporation and an electroless plating technique and the surface of the polymer was coated with iron oxide (FeO) particles. Structural characterization was performed using Optical Microscopy (OM), Scanning Electron Microscopy (SEM), Fourier Transform Infrared spectroscopy (FTIR), Energy Dispersive X-ray diffraction (EDAX), and Thermogravimetric Analysis (TGA). A study on the effect of the varying initial concentration of arsenic ions on percentage removal was performed in the laboratory and the adsorption capacity of the adsorbent was measured. Adsorption isotherm studies were carried out to evaluate the adsorption efficiency of FHM in removing arsenic from contaminated water. The Langmuir and Freundlich isotherm models were used to analyze the equilibrium experimental data. The isotherm study revealed that Langmuir adsorption data are well fitted and the maximum adsorption capacity of FHM in removing arsenic is 10.031 mg g^−1^. This high arsenic uptake capability combined with a low density of FHM makes it a potential material for arsenic removal particularly during the fabrication of lightweight portable water purification devices.

## Abstract 2

Adsorption of arsenic onto iron-based adsorption media has been established as a convenient method for the removal of arsenic from contaminated water.

## Graphical abstract

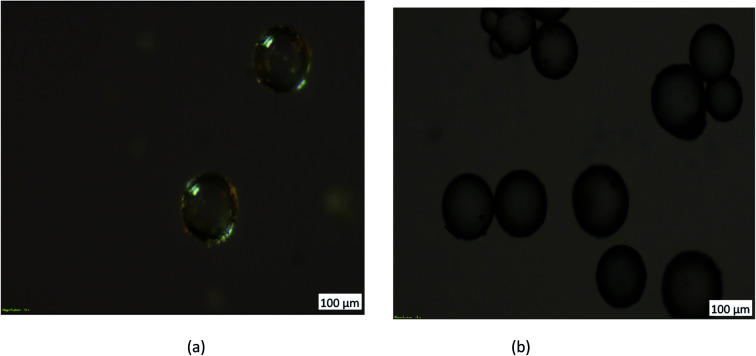

## 1 Introduction

Iron-based sorbents, which are also innocuous, inexpensive, chemically stable, and readily accessible, possess strong arsenic removal efficiency from drinking water.^2–7^ Thus iron(ii) oxide has a better sorption affinity towards As(v) or arsenates and As(iii) or arsenite, which are also electron-pair donors. Even iron oxide adsorbent systems possess a strong affinity for arsenic under neutral pH (pH ∼ 7) conditions. As(v) and As(iii) species form a coordinate bond becoming adsorbed on the surface of iron oxide.^2,8,9^ Since As(v) or As(iii) species possess ligand characteristics^10^ (electron-donating ability) and iron oxide has a larger surface area per unit mass and hence more sorption sites, selective separation of As(v) and As(iii) oxyanions or oxyacids from drinking water sources is highly facilitated.

However, despite the high sorption affinity of these submicron iron oxide particles, the stability of these particles and their aggregates in fixed beds is relatively low because of excessive pressure drops and poor mechanical strength.^7^ Therefore, embedding these particles into macroporous polymeric materials or any other host materials having the ability to bind effectively the metal oxide particles helps to overcome such drawbacks. Such examples of host materials are already available in literature which includes alginate^11,12^ zeolite,^13,14^ metal–organic framework,^15,16^ activated carbon,^2,6,17^ chitosan,^18–20^*etc.* In yet another example hydrated iron(iii) oxide nanoparticles were dispersed within a macroporous polymeric cation exchanger to develop a hybrid material for arsenic removal.^7,21^ Other techniques used for the removal of arsenic(iii) ions from water is coagulation,^22–24^ adsorption,^19,25,26^ and reverse osmosis.^27–29^ Except for adsorption which is an effective and economical method, other methods have high operational cost and less efficient for arsenic removal.^30,31^ Several studies on the adsorption of arsenic(iii) have been carried out using various adsorbents, such as fly ashes,^32–34^ natural and synthetic clay materials,^35,36^ ion-exchange resins,^21,37,38^ carbon nanotubes^39,40^ and metal oxides.^1,41,42^ Additionally, polymeric adsorbents in the form of hollow microspheres possess advantages such as low density, high surface area with a size range of about 1 to 1000 μm, and can readily be synthesized using various polymerization techniques.^28,43^ Their performance can also be optimized for targeted applications by coating the surface^29^ with specific materials. Therefore, these adsorbents are used for the removal of a wide variety of contaminants from drinking water.^30–34^ Arsenite, As(iii), and arsenate, As(v) are the predominant oxidation states of arsenic found in water, under reduced and oxygenated conditions respectively,^25,44^ and are potentially harmful to health. Trace amounts of methylated arsenic species are typically found in drinking water, and higher levels are found in biological systems.^45^ The concentration of arsenic in open ocean seawater and groundwater is 1–2 μg L^−1^, although groundwater concentrations can be up to 3 mg L^−1^ in areas with volcanic rock and sulfide mineral deposits.^46^ A high level of arsenic contamination in drinking water^47^ has been reported in several parts of the world which include the countries like Bangladesh, China, West Bengal, Australia, *etc.*^44^ In most of these regions, the drinking-water source is groundwater, naturally contaminated with arsenic-rich geological formations. Long-term exposure to arsenic from drinking-water and food can cause melanosis, edema, keratosis, dark spots on the chest, enlargement of liver, kidney, and spleen, cancers of the skin, lungs, and urinary bladder, *etc.*^48–52^ As per WHO recommendations, arsenic contamination should be less than 0.05 mg L^−1^ in drinking water while the same recommended by BIS is less than 0.01–0.05 mg L^−1^.^53^ Hence arsenic removal from drinking water sources has become a major concern nowadays for individuals at the household level and water distribution companies as well.

The present study thus emphasizes the supremacy of iron oxide coated hollow (polymethylmethacrylate) microspheres as adsorptive media for removal of arsenic from drinking water. The effects of the operational parameters, such as initial adsorbate concentration, contact time, and pH have also been investigated.

## 2 Materials and methods

### 2.1 Materials

Polymethylmethacrylate (PMMA) was purchased from Sigma-Aldrich with an average molecular weight of about 120 000 Da, and 98% viscosity 0.20 dL g^−1^ (lit.), dichloromethane 99.5% was procured from Merck with [*M* = 84.93 g mol^−1^], poly vinyl alcohol was obtained from Central Drug House, Delhi, MW (Avr.) 125 000 Da and 99.25% viscosity 35–50 cP at 4% cold aqueous solution. Stock solutions of 1000 μg ml^−1^ iron were prepared from FeSO_4_·7H_2_O [Merck, 99%, *M* = 278.02 g mol^−1^] using double distilled water. All other reagents used were of analytical grade and were obtained from Merck, India. Limestone was obtained from a mining site, Meghalaya, India.

### 2.2 Preparation of PMMA microsphere

Hollow PMMA microspheres were synthesized by solvent evaporation technique.^54^ A solution was prepared by dissolving PMMA (5–6% w/v) in dichloromethane with occasional stirring. The resultant solution was then added dropwise to a stirring aqueous medium. The aqueous medium comprises (0.5%, w/v) of poly(vinyl alcohol) which acts as a stabilizer. The stirring was maintained at 550 rpm with a propeller-type mechanical stirrer. Hollow PMMA microspheres (HPMM) were formed by slow evaporation of dichloromethane at room temperature. The material so obtained was washed with water and dried at 70 °C. The bulk density of the HPMMwas calculated to be 0.69 gm cm^−3^.

### 2.3 Preparation of iron oxide coated HPMM (FHM)

An electroless coating process, a literature procedure used for polyaniline coating on HPMM surface, was used for iron oxide coating on PMMA. Hollow PMMA (2% w/v) microspheres obtained above were mixed for 10 min in a 200 mg L^−1^ iron solution. The pH of the solution was maintained at 10 with the help of a 0.5 N NaOH solution. The iron oxide-coated PMMA microspheres (FHM) were then filtered and washed several times with distilled water until the runoff was clear. Then the mixture was dried at 65 °C and stored in capped bottles. The bulk density of the FHM was calculated as 1.27 gm cm^−3^.

### 2.4 Characterization of materials

The synthesized materials were characterized by using a scanning electron microscope having energy dispersive X-ray analysis attachment (Carl ZEISS, EVO50), FTIR (Bruker model Alpha-T), Optical microscope with Leica DMLM/P, Leica Microsystems AG Switzerland at 50× magnification, and Thermogravimetric analysis (TGA) (TA Instrument USA, Model 2950 and 2910). The specific surface area and porosity were estimated through the Brunauer–Emmet–Teller (BET) method using the 3 Flex instrument (Micromeritics). FT-IR of the samples was taken with the help FT-IR spectrophotometer (Bruker Alpha-model with KBr). The UV-Visible spectrophotometer is a double beam spectrophotometer from ANALYTICA JENA MODEL SPECORD 205. The testing for iron content in water was carried out by using standard method IS: 3025 (part 53).^53^

## 3 Results and discussion

### 3.1 Characterization of HPMM and FHM

#### 3.1.1 Surface morphology study

**Fig. 1:**
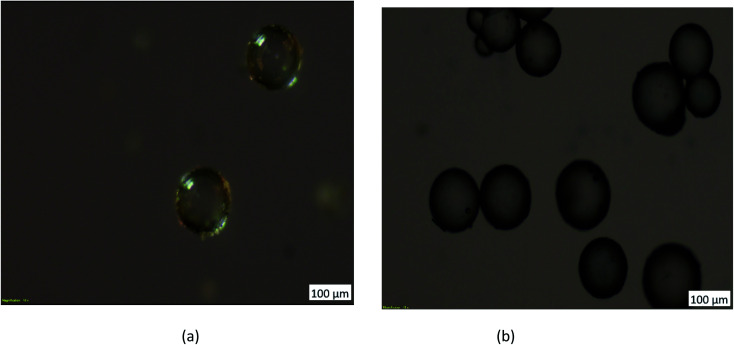
Optical microscope analysis of HPMM & FHM.

The optical micrographs of uncoated HPMM and iron oxide-coated HPMM are shown in Fig. 1. The uncoated HPMM appear as spherical white colored spheres of varying sizes in the micrograph while the iron oxide coated microspheres appear as brown/dark brown due to deposition of FeO on the surface. The observed color change in the case of FHM clearly indicates the coating of iron oxide onto the surface of HPMM.

**Fig. 2:**
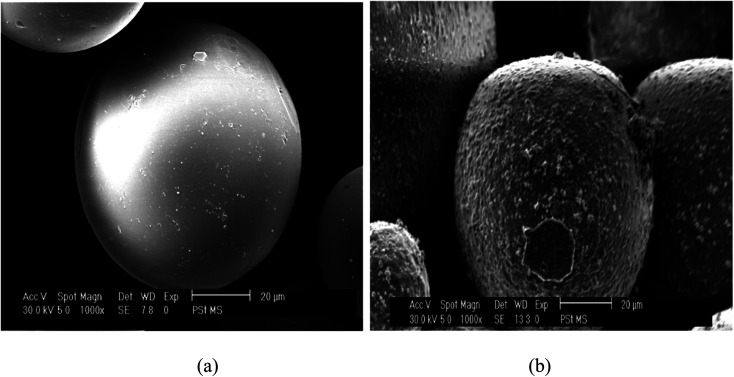
SEM analysis of HPMM & FHM.

Coating of iron oxide onto the surface of HPMM was further revealed from the images recorded during the morphological study with a scanning electron microscope (SEM) having energy dispersive X-ray analysis attachment (Carl ZEISS, EVO50). The images as shown in Fig. 2 display the difference in surface topography of HPMM and FHM producing conclusive evidence in support of iron oxide coating on to the surface of HPMM. The diameter of HPMM was found in the range of 20–80 μm from the SEM micrographs. The micrographs of HPMM show clearly the smooth surface of microspheres, whereas the surface of FHM shows the presence of precipitate of FeO as is evident from the appearance of a rough surface on the microsphere. The Brunauer–Emmet–Teller (BET) approach was used to measure the real surface area and porosity using the 3 Flex instrument (Micromeritics). BET is a surface characterization approach based on the physical absorption of gas molecules in a monolayer shape on a solid surface under pressure. The specific surface area (*S*) and porosity (*ε*) of the particles can be measured using data from the system's relative pressure and the density of the gas molecules being absorbed. The was observed that the real surface area (*S*) is 8.6 m^2^ g^−1^, and the porosity (*ε*) is 18.5 percent which decreases to real surface area (*S*) is 3.8 m^2^ g^−1^, and the porosity (*ε*) is 5.8 percent after the arsenic removal process.

#### 3.1.2 EDAX study

**Fig. 3:**
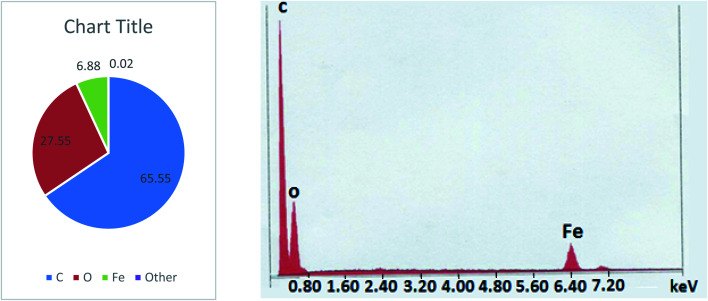
EDAX analysis of FHM.

The EDAX was carried out in a scanning electron microscope with energy dispersive X-ray analysis attachment using liquid nitrogen. The result was shown in Fig. 3. The results showed 6.88% of the iron coating in the synthesized product along with 65.56% carbon and 27.55% oxygen. The same has been confirmed taking multiple spot reading and area reading.

#### 3.1.3 TGA study

**Fig. 4:**
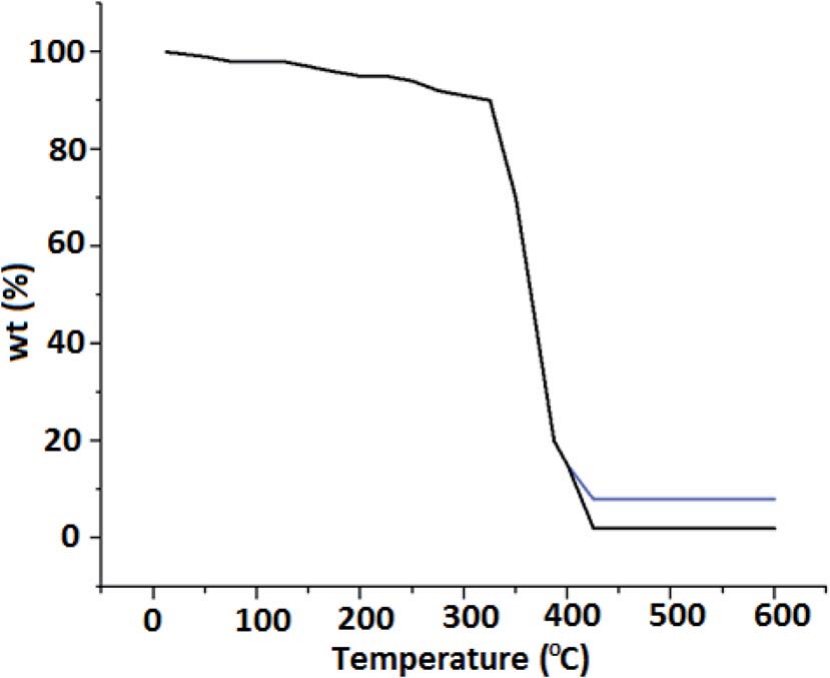
TGA spectra of HPMM (blue) & FHM (red).

Thermogravimetric analysis (TGA) was carried out in the non-inert atmosphere. The results although exhibited a similar pattern as the standard PMMA sample, but a clear difference was observed for the residual weight after 400–425 °C as shown in Fig. 4. From Fig. 4, it is clear that the major decomposition region for both samples is from 225 °C to 420 °C. Most of the polymeric core gets decomposed upto 420 °C. The residual weight fraction of FeO in FHM was observed to be 7.25%. This is in variation to the energy dispersive X-ray analysis study showing 6.88% of the iron coating in the synthesized product. This may be due to uneven coating. This is a favorable incidence as compared to even surface area, the uneven coating provides more surface area. More surface area results in more interaction with the contaminants in aquatic media and hence more effective removal of contaminants from water bodies.

#### 3.1.4 FTIR study

**Fig. 5:**
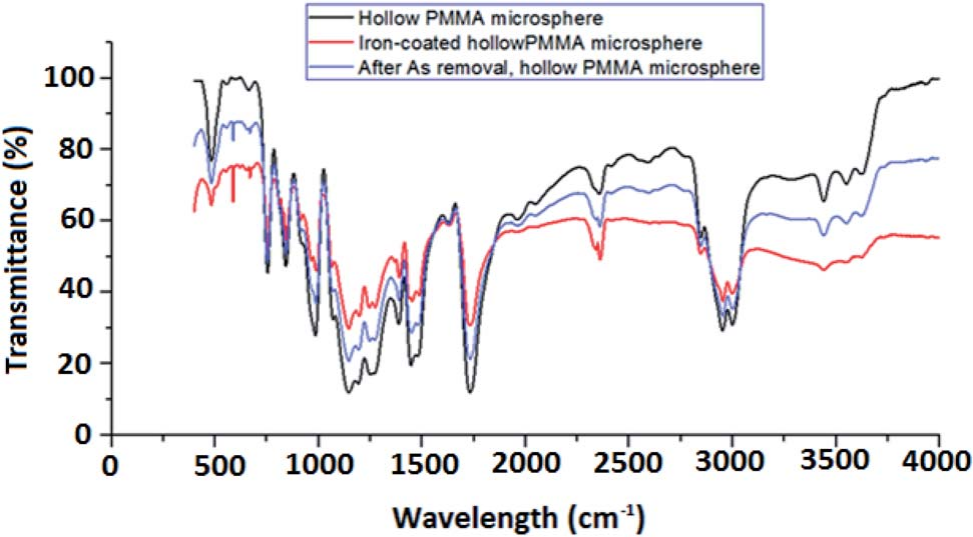
FTIR spectra of HPMM (blue) & FHM (red).

FTIR spectra of HPMM and FHM are depicted in Fig. 5. From the figure it can be seen that there is a distinct absorption band from 1150 cm^−1^ to 1250 cm^−1^, which can be attributed to the C–O–C stretching vibration. The two bands at 1388 cm^−1^ and 750 cm^−1^ can be attributed to the α-methyl group vibrations. The band at 987 cm^−1^ is the characteristic absorption vibration of PMMA, together with the bands at 1062 cm^−1^ and 845 cm^−1^. The band at 1740 cm^−1^ shows the presence of the acrylate carboxyl group. The band at 1440 cm^−1^ can be attributed to the bending vibration of the C–H bonds of the –CH_3_ group. The two bands at 2998 cm^−1^ and 2942 cm^−1^ can be assigned to the C–H bond stretching vibrations of the –CH_3_ and –CH_2_– groups, respectively. Furthermore, there are two weak absorption bands at 3437 cm^−1^ and 1648 cm^−1^, which can be attributed to the –OH group stretching and bending vibrations, respectively, of physisorbed moisture. However in the case of FHM an additional peak is observed at 587 cm^−1^ which can be assigned due to Fe–O stretching vibration, confirming the coating of iron oxide particles onto the surface of HPM.

#### 3.1.5 XRD study

**Fig. 6:**
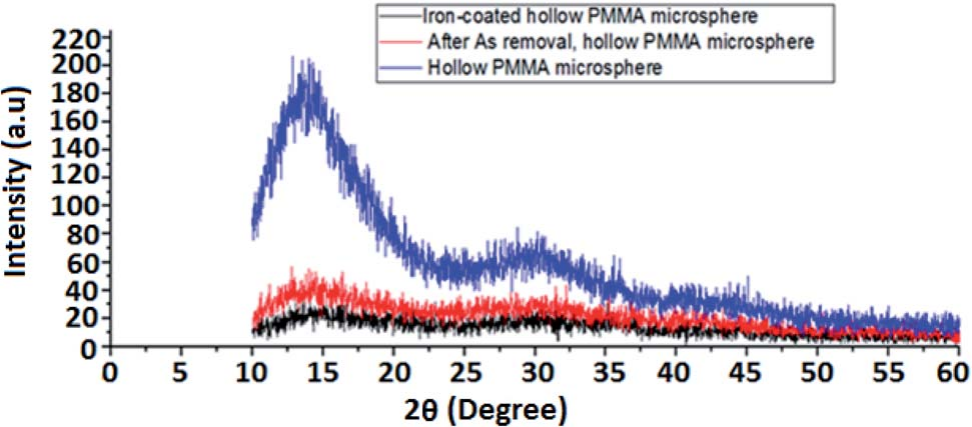
XRD patterns of HPMM and FHPM.

The XRD pattern of HPMM and FHPMM is shown in Fig. 6. A wide and shallow peak can be seen at around 2 of 16°, which is a characteristic of amorphous PMMA. The other two peaks, at 30.50° (220) and 43.46° (400), can be due to normal diffraction of surface coated FeO particles, suggesting that FeO was successfully coated on the polymer surface. The concentration of iron oxide is extremely low, at just 5%. The diffraction peaks are very small due to the low concentration of iron oxide and its presence in combination with PMMA. Tao Chen^55^ had explained the phenomenon in the case of PMMA/Fe_2_O_3_ composite.

### 3.2 Optimization of adsorption parameters

The adsorption kinetics reflects the characterization of the arsenic removal process as a function of time and concentration. Optimization of different adsorption parameters such as contact time of the adsorption process, As(iii) concentration, and pH was effectively carried out as it plays a significant role in determining the type and the number of adsorbents that one should use for the process to take place efficiently.

#### 3.2.1 Effect of As(iii) concentration on percentage removal and adsorption capacity

**Fig. 7:**
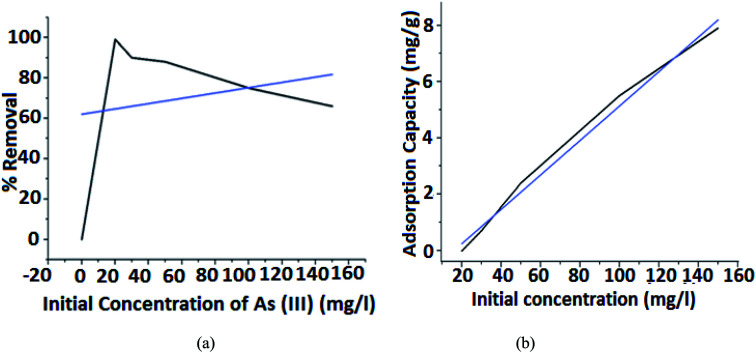
Effect of initial concentration of As(iii) on (a) % removal and (b) adsorption capacity.

Experiment on iron-coated PMMA was conducted with an initial As(iii) concentration of 20 mg L^−1^, 30 mg L^−1^, 50 mg L^−1^, 100 mg L^−1^ and 150 mg L^−1^ at five different contact times and the percentage of removal was estimated to be 99.1%, 90.4%, 88%, 75%, and 66% respectively. Experimental results are displayed in Fig. 7.

In Fig. 7(a), it is observed that a high percentage of 99.1% is achieved for an initial concentration of 20 mg L^−1^ and the percentage of removal decreased with the increase of the initial As(iii) concentration. This is because the number of available active sites for adsorption in FHM is less compared to the available metal ion in the medium. These variations are small at low concentrations because of the smaller ratio of the initial number of metal ions to the available adsorption active sites. However, it becomes more significant with the increase in the initial concentration.

From Fig. 7(b), it is observed that for 10 mg L^−1^ of arsenic concentration, the absorption capacity is 1.9833 mg g^−1^ whereas for 30 mg L^−1^ of arsenic concentration the capacity increases to 2.701233 mg g^−1^ indicating that the equilibrium adsorption capacity for As(iii) ion increases with an increase in the concentration of As(iii) ions. Therefore, it is assumed that at low initial concentrations, the monolayer is formed at the outer surface of the adsorbent and controls the adsorption rate. Thus, the adsorption process is very intense and fast, as most adsorbent active sites remain unsaturated.

### 3.3 Adsorption isotherms

Adsorption studies allow us to investigate the distribution of adsorbate and adsorbent in the solution at equilibrium conditions in the form of adsorption isotherms. These isotherm plots establish the relationship between the amounts of adsorbed and non-adsorbed quantities during series of adsorption at a given temperature and pressure. From these plots, we can also obtain the nature of the adsorption process. The distribution of metal ions between the liquid phase and the solid phase can be described by several isotherm models such as Langmuir and Freundlich. These models are used to determine the efficiency of synthesized FM in removing As(iii) ions from contaminated water.

#### 3.3.1 Langmuir isotherm

The adsorption isotherm is an important curve depicting the marvel overseeing the intake or portability of a substance from the fluid permeable media or amphibian conditions to a strong stage at a steady temperature and pH. Throughout the years, a wide assortment of harmony isotherm models (Langmuir, Freundlich, Brunauer–Emmett–Teller, Redlich–Peterson, Dubinin–Radushkevich, Temkin, Toth, Koble–Corrigan, Sips, Khan, Hill, Flory–Huggins and Radke–Prausnitz isotherm), have been formulated. Langmuir adsorption isotherm, initially created to portray gas-strong stage adsorption onto activated carbon, has generally been utilized to evaluate and differentiate the exhibition of various bio-sorbents. In its details, this exact model expects monolayer adsorption (the adsorbed layer is one particle in thickness), with adsorption, can just happen at a limited (fixed) number of distinct confined destinations, that are indistinguishable and equal, with no parallel cooperation and steric obstacle between the adsorbed atoms, even on adjoining locales. In its determination, Langmuir isotherm alludes to homogeneous adsorption, in which every atom has consistent enthalpies and sorption initiation vitality (all locales have an equivalent proclivity for the adsorbate), with no immigration of the adsorbate in the plane of the surface.

The Langmuir adsorption isotherm is used to describe the equilibrium between the adsorbate and the adsorbent system, where the adsorption partial pressure approaches saturation.^56^ This means that this isotherm is suggested when the adsorbate occupies a site where further adsorption cannot take place. All sites are energetically equivalent and there is no interaction between molecules adsorbed on neighboring sites.^57^

The Langmuir equation is written in a linear form as follows:

1 *q*_e_/*C*_e_*= K*_L_*q*_m_*− K*_L_*q*_e_

whereat equilibrium conditions, *q*_e_ (mg g^−1^) is the amount of As(iii) adsorbed, *C*_e_ (mg L^−1^) is the concentration of the As(iii) solution, *K*_L_ is the Langmuir constant related to adsorption enthalpy *q*_m_ is the maximum adsorption capacity.

**Fig. 8:**
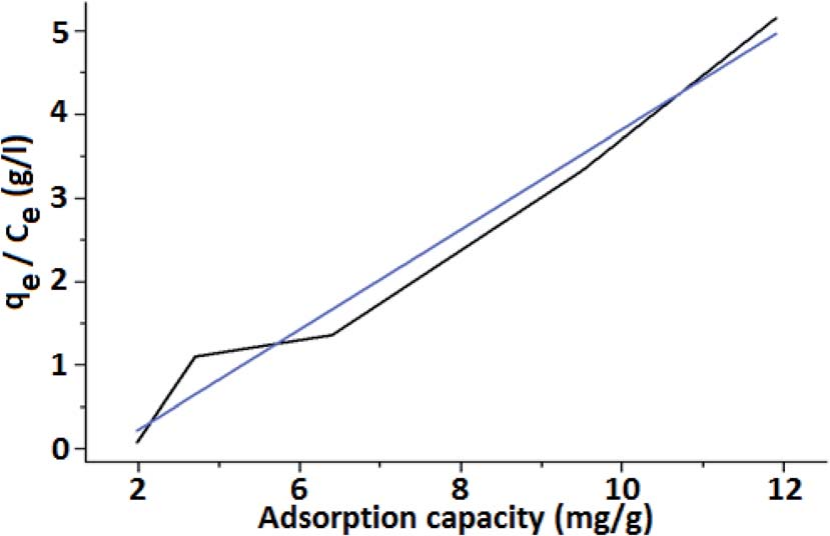
Langmuir adsorption isotherm.

Fig. 8 shows the Langmuir isotherm model using FHM to remove arsenic from water. The obtained linear plot indicates the monolayer coverage of the material. The adsorption capacity (*q*_e_) is the amount of adsorbate (arsenic) that a particular adsorbent (iron coated PMMA) is capable of removing. It is characterized by the relation:

2 *q*_e_ = *X*/*M*

where, *X* = amount of adsorbate (arsenic in water). *M* = mass of adsorbent (iron oxide coated PMMA).

Maximum adsorption capacity (*q*_m_) is the capacity to retain the maximum amount of an adsorbate (arsenic) per unit mass of the adsorbent (FHM). In general, the adsorbent achieves *q*_m_ at a lower concentration, and rarely it is obtained at higher concentrations. The adsorption enthalpy is correlated with Langmuir isotherm constant (*K*_L_ in L mg^−1^). The isotherm constant *K*_L_ is the affinity of FHM towards the adsorbate (arsenic in this case). From eqn (1), the slope is obtained as *K*_L_ and the intercept is *q*_m_. The correlation coefficient *R*^2^ value is used to indicate whether the adsorption is favorable or whether a better correlation of parameters is required. From the results of the experiment carried out with FM, it was observed that the maximum adsorption capacity (*q*_m_) for FHM was achieved at around 10.031 mg g^−1^. The obtained value of the Langmuir constant (*K*_L_) is 1.478 L mg^−1^ and the value of *R*^2^ is calculated as 1. Experimentally obtained adsorption data of the Langmuir isotherm & Freundlich isotherm parameters are listed in Table 1.

**Table 1:** Langmuir isotherm & Freundlich isotherm parameters

Langmuir isotherm			Freundlich isotherm			
*q*_m_ (mg g^−1^)	*K*_L_ (L mg^−1^)	*R* ^2^	1/*n*	*n*	*K*_F_ (L mg^−1^)	*R* ^2^
10.031	1.478	1	0.280	3.559	3.280	0.902

Langmuir model is an empirical model having a linear plot, which indicates that the active sites on the surface are filled linearly and the monolayer of FHM is fitted into the heterogeneous surface. The *R*_L_ is a separation factor and its value is used to evaluate the characteristic of the Langmuir isotherm. The *R*_L_ value is inversely proportional to *q*_e_ and it decreases with the increase in the adsorption capacity (*q*_e_). Thereby, the isotherm process is favored.

3 *q* = 1/[1 + (*R*_L_ + *C*_o_)]

**Fig. 9:**
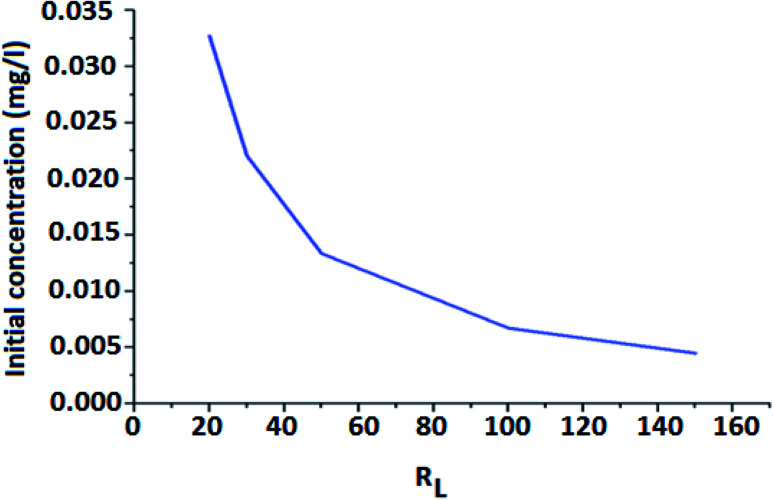
Langmuir isotherm for adsorption of As(iii) ion on FeO coated PMMA surface.

From Fig. 9, it is revealed that the value of *R*_L_ is dependent on the initial concentration of the adsorbents. It is seen that separation factor *R*_L_ decreases with increasing concentration from 0.032 at 20 mg L^−1^ to 0.022 at 30 mg L^−1^. As the value of *R*_L_ is less than 1, it indicates that adsorption is favorable.^58^

#### 3.3.2 Freundlich isotherm

Freundlich isotherm is an empirical model describing adsorption onto a heterogeneous surface and suggests multilayer adsorption.^59^ The energy of adsorption decreases exponentially on completing the filling of active sites of an adsorbent. The linear form of the Freundlich isotherm is shown in eqn (4).

4 log *q*_e_ = log *K*_F_ + (1/*n*)log *C*_e_

where ‘*K*_F_’ and ‘*n*’ are Freundlich constants related to the adsorption capacity and the adsorption intensity of the adsorbent respectively. 1/*n* is the heterogeneity factor and ‘*n*’ is a measure of the deviation from the linearity of adsorption.

The adsorption capacity (*K*_F_) and the adsorption intensity (1/*n*) are directly obtained from the slope and the intercept of the linear plot of log *q*_e_*versus* log *C*_e_. The higher fractional values of 1/*n* signify that strong adsorption forces are operative on the system. The magnitude of 1/*n* also indicates the favorability and capacity of the adsorbent/adsorbate system. The value 1/*n*, between 0 and 1, represents favorable adsorption.

**Fig. 10:**
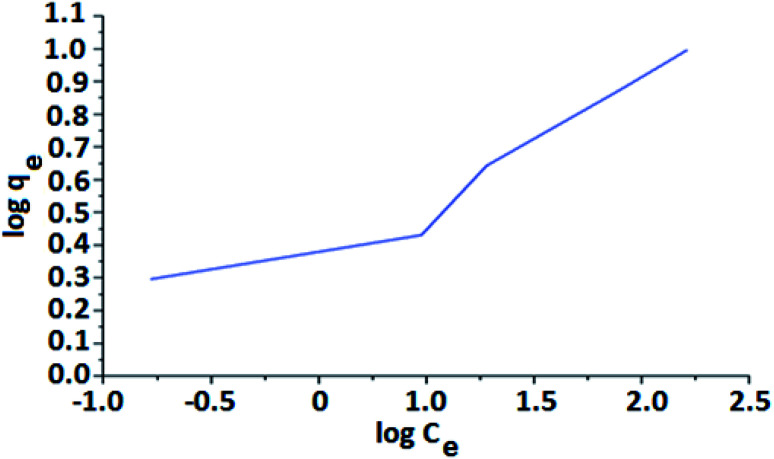
Freundlich isotherm as a plot of log *q*_e_ against log *C*_e_.

Fig. 10 shows Freundlich isotherm as a plot of log *q*_e_ against log *C*_e_. The plot displays a deviated line with a slope of 1/*n* and intercept of log *q*_m_. The 1/*n* is the Freundlich constant related to sorbent intensity, whereas *q*_m_ is the Freundlich constant associated with adsorption capacity.

The adsorption of As(iii) metal ions on FHM is described by Langmuir and Freundlich isotherms. Based on the *R*^2^ values, we can mention that Langmuir isotherm provided a better fit compared to Freundlich isotherm. This fact otherwise suggests that maximum adsorption of As(iii) metal ions occurred *via* monolayer of the adsorbate.

The magnitude of the Freundlich adsorption capacity ‘*n*’ indicates favourability of adsorption. The values of ‘*n*’ ranges from 2–10 indicating good adsorption capacity, 1–2 moderate adsorption capacity, and less than one indicates poor adsorption capacity. 1/*n* is a function of the strength of the absorbent material. The smaller value of 1/*n* (<1) implies stronger interaction and the greater value of 1/*n* (>1) implies weaker interaction between adsorbate and adsorbent.^60^ Also, the absorption coefficient increases with an increase in the concentration of the solution and that eventually led to an increase in hydrophobic surface characteristics after monolayer. While 1/*n* equals 1 indicates linear adsorption sites leading to identical adsorption energies for all sites. The *n* value obtained from the curve (which is the measurement of the favorability (0 < *n* < 10) of adsorption) is 3.55. In general, the value of *n* is greater than 1 due to the distribution of surface sites, leading to the decrease in the adsorbent–adsorbate ratio in the case of higher surface density. As a result, the adsorption process is favorable when *n* > 1. The value of *K*_F_ denotes the affinity of the adsorbent towards the adsorbate molecules. The *K*_F_ value, in this case, is 3.280789 L mg^−1^. The high value of *K*_F_ indicates more binding of adsorbate molecules on the surfaces of the adsorbent. The coefficient of correlation (*R*^2^) denotes the favorability and the fitting of the Freundlich data onto the modeling analysis. In the case of FHM, the *R*^2^ was observed to be 0.9027 which is near to the Langmuir correlation factor (*R*^2^ = 1).

Freundlich isotherm is an empirical construct. So the logarithm data fits the equations and the obtained value of *R*^2^ validates that the modeling is better in the case of the Langmuir isotherm model and the dataset fits well into it. The reason for this is the high separation factor value obtained through the Langmuir isotherm model.

Table 2 presents the comparison data for arsenic removal, carried out by different researchers.

**Table 2:** Comparison of adsorption capacities of different adsorbents for arsenic

S.No	Adsorbents	Adsorption capacity (mg g^−1^)		References
		As(iii)	As(v)	
1	Iron oxide coated hollow PMMA	8.12	10.03	Present study
2	Granular ferric hydroxide (GFH)	—	1.1	^61^
3	Ultrafine δ-FeOOH	—	37.3	^62^
4	Magnetite–maghemite nanoparticles	3.69	3.71	^63^
5	α-Fe_2_O_3_	—	0.2	^64^
6	Fe_3_O_4_ nanoparticles	16.56	46.06	^65^
7	g-Fe_2_O_3_ nanoparticles	—	2.9	^66^
8	Fe_3_O_4_-γ-Fe_2_O_3_ nanoparticles	3.69	3.71	^67^
9	Bituminous based Filtrasirb 400	—	2.45	^68^
10	Modified activated carbons with iron hydro(oxide) nanoparticles	0.035	(Initial total As conc. is 0.31 mg L^−1^)	^69^
11	Lignite-based AC	—	0.26 (initial As conc. is 0.12 mg L^−1^)	^70^
12	Ferric oxyhydroxides anchored onto activated carbon	26.8	—	^71^
13	Straw activated carbon	51.3	33.8	^72^
14	Iron-impregnated granular activated carbon	—	1.95 (initial As conc. is 0.12 mg L^−1^)	^73^
15	Sawdust-based AC	—	204	^74^
16	Fe_3_O_4_ coated wheat straw	3.9	8.1	^75^
17	ZVI nanoparticles modified starch	12.2	14	^76^
18	Iron loaded orange peel	68.2	68.6	^69^
19	Coconut shell with 3% ash	—	2.4	^77^
20	Ce–Ti oxide adsorbent	6.8	7.5	^78^
21	Char carbon	89	34.46	^79^
22	Activated bauxsol (red mud)	0.541	7.642	^80^
23	Empty fruit bunch biochar	18.9	5.5	^81^
24	Leonardite char	4.46	8.4	^82^
25	Magnetic Fe_3_O_4_ nanoparticles (tea waste)	189	154	^83^

**Fig. 11:**
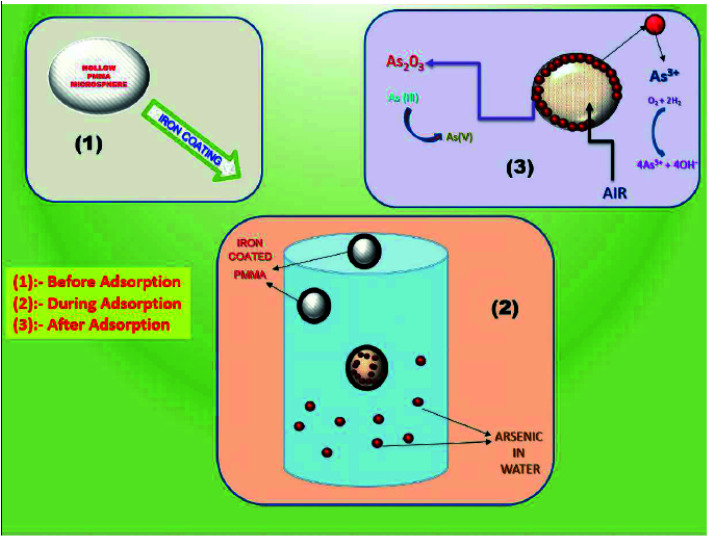
Mechanism of arsenic removal by FHM.

The process of removal of arsenic from the water is shown in Fig. 11. When the iron-coated HPMM are brought in contact with the arsenic-contaminated water solution, the arsenic intends to deposit on the surface of FHM. In this case, the FeO layer on the polymeric surface acts as a catalyst surface and facilitates the oxidation of arsenic ions present in the aqueous medium. Therefore, arsenic adsorbed on the surface of FHM being converted to arsenic trioxide form, which is further separated from the solution. Thus, the arsenic is removed from the drinking water by an iron coating layer on FHM.

**Fig. 12:**
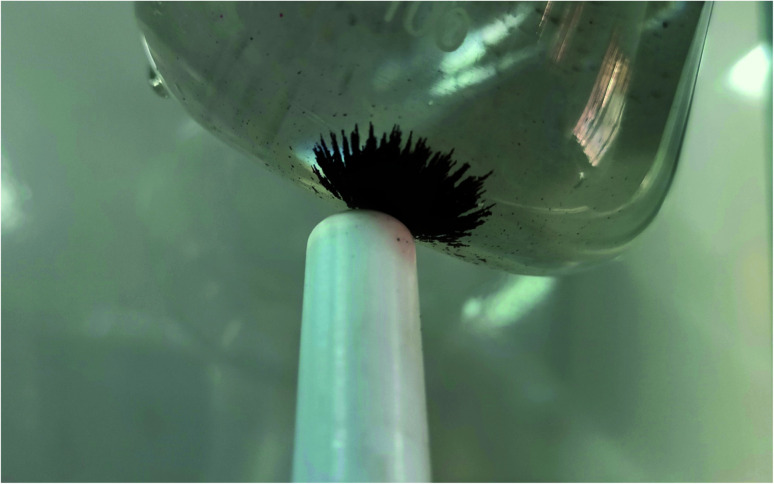
Magnetic recovery of the adsorbent.

A regeneration study on the efficiency of adsorbent is carried out too. A mild acidic solution (pH < 5) is used to wash the adsorbent after the study. The absorbance study has retreated on the same adsorbent. The recovery rate is calculated as 98.2% after 10 continuous studies. The adsorption of arsenate and arsenite increases as pH becomes more alkaline, because the positive charges on the iron cations attract the negative charges of the arsenic anions, creating ionic bonds. The lowering pH retracts the phenomenon causing recovery of the adsorbent. The adsorbent can be recovered easily by using any magnetic retriever as shown in the Fig. 12.

## 4 Conclusion

We have successfully demonstrated the synthesis of iron oxide-coated hollow polymethylmethacrylate microspheres, which are highly efficacious in removing arsenic(iii) from drinking water. The material (FHM) also possesses other advantages such as low density, high surface area, and economic viability. The arsenic removal efficiency is influenced by an operational parameter such as the concentration of arsenic ions in the solution. The removal efficiency of ‘As’ is high at low concentrations and decreases as the concentration of ions increases in the solution. Adsorption studies performed by using Langmuir and Freundlich adsorption isotherm models showed that the Langmuir isotherm model is well fitted into the adsorption data of arsenic ions. Overall from the ease of synthesis and economic aspect, we can conclude that the FHM can be projected as a viable material for the removal of arsenic from drinking water, which otherwise provides a solution to a major environmental and health concern.

## Ethics approval and consent to participate

Ethics approval has taken whenever necessary and consent to participate all participants are also obtained.

## Consent for publication

Consent for publication is taken from the competent authority and all authors.

## Availability of data and material

All data is made available in manuscript.

## Conflicts of interest

There are no conflicts to declare.

## Funding

No funding has been received from any source for this manuscript.

## Author contributions

DD performed the material synthesis and wrote the manuscript, JPB facilitated the infrastructure and provided the necessary guidance for the article, AP performed final editing of the article and overall guidance.

## Supplementary Material
